# Sociodemographic and Health-Related Factors Influencing Drug Intake among the Elderly Population

**DOI:** 10.3390/ijerph19148766

**Published:** 2022-07-19

**Authors:** Alicja Pietraszek, Siddarth Agrawal, Mateusz Dróżdż, Sebastian Makuch, Igor Domański, Tomasz Dudzik, Krzysztof Dudek, Małgorzata Sobieszczańska

**Affiliations:** 1 Clinical Department of Geriatrics, Wroclaw Medical University, Skłodowskiej-Curie Str. 66, 50-369 Wroclaw, Poland; malgorzata.sobieszczanska@umw.edu.pl; 2 Department and Clinic of Internal Medicine, Occupational Diseases, Hypertension and Clinical Oncology, Wroclaw Medical University, Borowska Str. 213, 50-556 Wroclaw, Poland; siddarth@agrawal.pl; 3 Faculty of Medicine, Wroclaw Medical University, Jana Mikulicza-Radeckiego 5, 50-345 Wroclaw, Poland; mateuszdrozdz5208@gmail.com (M.D.); igor.domanski@student.umw.edu.pl (I.D.); tomasz.dudzik@student.umw.edu.pl (T.D.); 4 Department of Clinical and Experimental Pathology, Wroclaw Medical University, K. Marcinkowskiego Str. 1, 50-368 Wroclaw, Poland; sebastian.mk21@gmail.com; 5 Faculty of Mechanical Engineering, Wroclaw University of Science and Technology, Str. I. Łukasiewicza 5, 50-371 Wroclaw, Poland; krzysztof.dudek@pwr.edu.pl

**Keywords:** sociodemographic factors, drug intake, elderly population

## Abstract

Excessive drugs intake among the elderly population, including self-medication, constitutes an important public health problem. Polypharmacy may lead to numerous adverse health effects, which become more prevalent when combined with biological changes in seniors. In this cross-sectional study, 500 Polish adults aged ≥60 years (M = 67.9 ± 4.2) were asked to complete a questionnaire via telephone calls, allowing us to identify sociodemographic and health-related factors influencing the daily medications consumption. Our findings revealed that all of the participants were receiving medications; 60.2% of them receive at least 1 to 3 drugs per day (301/500). The most commonly used medications included antihypertensive drugs and analgesics (51.0% and 46.0%, respectively). Taking into account clinical conditions, independent predictors of receiving over 3 medications per day turned out to be (1) coronary artery disease (OR = 6.77; CI 95%, 2.86–16.1), (2) diabetes (OR = 3.23, CI 95%, 1.75–5.95), (3) asthma (OR = 4.87, CI 95%, 2.13–11.1), (4) heart failure (OR = 3.38, CI 95%, 1.59–7.19) and (5) gastroesophageal reflux disease (OR = 1.93, CI 95%, 1.03–3.62). Participants suffering from depression were more likely to take drugs for hypertension (OR = 1.70, CI 95%, 1.04–2.78), while those with anxiety and social loneliness took more painkillers (OR = 2.59, CI 95%, 1.58–4.26 and OR = 2.08, CI 95%, 1.38–3.13, respectively). The most significant sociodemographic factors increasing the drugs intake among the population included in our study were high body mass and subsequent increased BMI values (OR = 2.68, CI 95%, 1.50–4.77). Furthermore, living in a city with over 400,000 inhabitants increased the likelihood of taking antidepressants (OR = 2.18, CI 95%, 1.20–3.94). Our study revealed factors increasing the risk of excessive medications intake and hence, increased susceptibility to some iatrogenic diseases among the elderly population. These factors should be considered by primary care physicians while prescribing appropriate drugs to elderly patients.

## 1. Introduction

Physicians taking care of elderly patients face many challenges resulting from the specificity of the geriatric population compared with younger adults. Alongside with increasing age, there is a higher prevalence of multimorbidity [1], which often implies a need for a more complex pharmacotherapy regimen. It is one of the reasons why the prevalence of polypharmacy, defined as routinely taking a minimum of five medications, including drugs prescribed by a doctor, bought over the counter (OTC) as well as traditional, herbal or complementary medicines [2], is increasing with age [3]. Polypharmacy constitutes a significant health problem among the elderly population. It is associated with many harmful effects, including adverse drug reactions, drug-drug reactions, higher mortality and fall rates, prolonged stay in a hospital, readmission to a hospital soon after discharge as well as increased healthcare costs and risk of medical nonadherence [4,5,6]. Moreover, aging is also related to changes in pharmacodynamic and pharmacokinetic properties of drugs, decreased renal and liver function, lower body and muscle mass, poor nutritional status, and lower hydration level, which puts the elderly patients at higher risk of experiencing adverse drug reactions [7].

Despite the increasing knowledge about adverse health consequences of a polypharmacy, some alarming data indicate that more than 40% or even more than half of the prescribed drugs may not have sufficient clinical justification [8,9]. It is worth mentioning that the problem is considered as the most significant among the oldest patients, aged 80 years and more [9]. Some drugs are also being prescribed as a part of a prescribing cascade, when an adverse drug reaction occurs and is misinterpreted as a new medical condition, resulting in a subsequent drug prescription to treat it [10]. Some examples of a prescribing cascade may include calcium channel blockers causing ankle oedema followed by prescribing diuretics or ACEI causing cough, treated with antitussives [11].

Another major challenge in pharmacotherapy in the elderly population is self-medication and usage of over-the-counter (OTC) dispensed drugs. According to Cybulski et al., most seniors buy OTC drugs, and more than 40% of seniors take one OTC drug regularly [12]. As those drugs may be purchased without a prescription, it makes them readily available to patients. However, these drugs may still interact with other medications or may be used incorrectly, causing severe adverse health effects.

The population of people over 60 is constantly growing, currently much faster than in recent years. According to WHO, in 2050 it will constitute 22% of the global population [13]. It emphasizes the need to provide high-quality specialistic care for the elderly and makes it a priority for health care systems in the upcoming years. This makes a need to deepen the knowledge and extend the research in this area more urgent. Therefore, our study aimed to define which medication groups, both prescribed by health practitioners as well as OTC drugs bought by patients without medical prescription, are used most commonly in the elderly population in Poland. We also managed to identify health-related and socioeconomic factors with the most significant impact on the usage of a higher number of medications in older people. We believe that these data will sensitize health care providers about the problem of proper pharmacotherapy in the elderly population and enable them to focus their efforts on revising the treatment of their patients, starting with those who are at the highest risk of having unproper treatment regimen.

## 2. Materials and Methods

### 2.1. Study Design

A cross-sectional study was carried out in November–December 2020 in Poland. We surveyed a representative sample of 500 adults, including 290 women (58%) and 210 men (42%), of age 60 and above (M = 68, SD = 4.2). The evaluated sample of the elderly population was provided by Biostat Sp. z o.o. and obtained by a stratified sampling per voivodeship demographic structure of Poland. Target quotas were set for age and gender in each of the geographical regions. All the participants were interviewed by computer-assisted telephone calls. The gross sample was 1250. The identity of a participant was confirmed at the beginning of the interview. Interviewers were adequately trained and prepared to ensure the equal and adequate quality of the interview. Moreover, all interviews were supervised by a specialist. A study coordinator additionally evaluated recorded conversations. The transcripts were not returned to participants for any comment and/or correction, nor were repeat interviews carried out. The duration of the interview ranged from 15 to 20 min. Participants provided their verbal consent at the beginning of the interview and were informed about the goal of the survey. No compensation was provided for participating in the study. The study was approved by the Bioethics Committee of Wroclaw Medical University.

### 2.2. Explanatory Variables

The questionnaire used in the study included questions regarding the respondent’s sociodemographic data (Table 1), mental and physical health conditions (Table S1), existing comorbidities and taking influenza vaccination in 2019 and 2020 (Table 2). Sociodemographic data included: (1) gender (male or female), (2) age (categorized as 60–64; 65–69; 70 and more), (3) place of residence (village; town less than 20,000 inhabitants; town between 20,000 to 100,000 inhabitants; town between 100,000 to 200,000 inhabitants; town between 200,000 to 400,000 inhabitants; town more than 400,000 inhabitants), (4) household size (living alone; living with a partner; living with a partner and children; living with a family, (5) education (primary, vocational, secondary, higher), (6) body weight (kg), (7) body height (cm) and (8) BMI (kg/m^2^). Patients were also asked for (9) household net income per person per month (in Polish currency-PLN, categorized as less than 500 PLN; 501–1000 PLN; 100–2000 PLN; 2001–3000 PLN; more than 3000 PLN; refusal to answer). Data allowing to determine the mental and physical health conditions among the elderly population involved in the study were collected based on specified and validated scales, including (1) Katz Activities of Daily Living Scale (ADL) [14], (2) Lawton Instrumental Activities of Daily Living Scale (IADL) [15], (3) Abbreviated Mental Test Score (AMTS) [16], (4) Geriatric Depression Scale (GDS-15) [17], (5) Geriatric Anxiety Scale (GAS-10) [18], (6) Lubben Social Network Scale (LSNS-6) [19], (7) Social Loneliness Scale (Gierveld Scale; GLS) [20] and (8) Mini Nutritional Assessment (MNA) [21]. Questions regarding chronic diseases included: coronary artery disease, diabetes mellitus, asthma, COPD, heart failure, kidney failure and gastroesophageal reflux disease.

### 2.3. Measures

An original questionnaire containing seven questions was used to evaluate pharmacological treatment among the representatives of elderly population in Poland (Table 3). We asked participants about (1) the number of medications taken (1–3; 4–6; 7–10; >10) and (2) which group do they belong to (hypertension drugs; diuretics; painkillers; anticoagulants; antidepressants). Furthermore, respondents were asked (3) if the same doctor prescribed all medication or not; (4) if no; how many (1; 2; 3; 4; 5 and more). We also wanted to know if the patient (5) informed his or her family doctor about all new medications taken and (6) bought drugs and/or supplements without a prescription; (7) if yes, which ones (painkillers; drugs for heartburn; herbal medications; vitamins; other). Based on the results obtained, independent predictors of using more medications in the elderly population were determined, using the logit models. In each section, we presented the multivariate logistic regression analysis of different drug groups and socioeconomic conditions as well as the clinical and mental characteristics of the surveyed respondents.

### 2.4. Statistical Analysis

Nominal qualitative (e.g., gender) and ordinal (e.g., age group) variables are presented in the contingency tables in the form of frequency (*n*) and proportion (%). Quantitative variables (e.g., BMI) with a distribution close to normal are presented in tables and graphs with mean and standard deviations (M ± SD). In cases where their distribution differed significantly from a standard (which was verified by the Kolmogorov Smirnov test), we presented these correlations in the form of medians and quartile ranges-Me (Q1–Q3).

Chi-square tests of independence were used to assess the significance of the correlation between the two qualitative variables. The significance of differences between the average values of quantitative variables in the two groups was assessed using the Mann-Whitney U test. The Kruskal-Wallis test was used for a more significant number of groups. For multiple comparisons (post-hoc tests), the Bonferroni correction was taken into account.

Continuous or step quantitative parameters were transformed into dichotomous variables. ROC curves and Youden’s index were used to determine cut-off values. For the established threshold values, the sensitivity and specificity were estimated.

The multivariate logistic regression analysis and the method of backward elimination were used to determine the parameters significantly correlating with the number of taken drugs greater than 3.

The quality of the model was assessed based on the statistics of the Hosmer-Lemeshow test and determination coefficients; the statistical significance of the entire model was checked using the likelihood ratio test (LR test), while the statistical significance of a specific variable in the model was based on Wald’s test.

All analyses were performed using the statistical software package Statistica. A *p*-value of <0.05 was considered to be statistically significant.

## 3. Results

The cross-sectional analysis included 500 participants–290 women (58%) and 210 men (42%) of age 60 and more (M = 67.9 ± 4.2). The response rate was equal to 40%. Most of the participants lived in a city between 20,000 to 100,000 inhabitants (136/500; 27.2%) and fewer in villages (110/500; 22.0%). Most respondents were relatively highly educated; only eight people had primary education (8/500; 1.6%). Based on the given measurements of body mass and height, we calculated all participants’ body-mass index (BMI) (M = 27.4 ± 4.6). According to the World Health Organization (WHO) report, this result shows respondents were slightly overweight [5]. Considering household income per person per month, five people earned less than 500 PLN (5/500; 1.0%), 24 people earned between 501 PLN and 1000 PLN (24/500; 4.8%), 188 people earned between 1001 PLN and 2000 PLN (188/500; 37.6%), 158 people earned between 2001 PLN and 3000 PLN (158/500; 31.6%) and 110 respondents earned more than 3000 PLN (110/500; 22.0%) per person per month. This result should be considered with caution, as due to the restrictions caused by a prevailing COVID-19 pandemic, many people had lost their jobs or had lowered salaries. Detailed data on the general characteristics of the surveyed people showing their sociodemographic data are presented in Table 1.

According to the ADL scale, most participants were fit (493/500; 98.6%). However, according to the GDS-15 scale, more than one-third of the study group showed depressive symptoms that indicated depression (176/500; 35.2%). According to the LSNS-6 scale, they exhibited proper social engagement (mean = 14.2 ± 5.9) and didn’t feel lonely (according to the Gierveld Scale (GLS), mean = 13.1 ± 1.8). According to the MNA scale, most of the participants had a proper nutritional status (418/500, 83.6%). Detailed data on the psychological characteristics of the surveyed people are presented in Table S1.

Most of the participants suffered from one or more chronic diseases, such as coronary artery disease (*n* = 63, 12.6%), diabetes mellitus (*n* = 74, 14.8%), asthma (*n* = 43, 8.6%), COPD (*n* = 33, 6.6%), heart failure (*n* = 71, 14.2%), kidney failure (*n* = 20, 4.0%) and gastroesophageal reflux disease (*n* = 68, 13.6%). Only 62 (12.4%) and 51 (10.2%) participants underwent influenza vaccination in 2019 and 2020, respectively. Such a low interest in vaccination was caused by a fear of possible vaccine adverse effects (*n* = 164, 32.8%) and lack of vaccines in pharmacies (*n* = 104, 20.8%). Moreover, the primary care physician recommended vaccination against influenza and pneumococci only in 81 (16.2%) participants. More than 50% of the study group knew about the flu vaccine reimbursement for seniors (259/500; 51.8%). Detailed data on the clinical characteristics of the studied people are shown in Table 2.

All of the participants were receiving medications. Most of them (*n* = 301, 60.2%) took 1 to 3 drugs, and 8 respondents used more than 10 drugs regularly (8/500; 1.6%). The most commonly used medications were antihypertensive drugs (*n* = 255, 51.0%) and analgesics (*n* = 230, 46.0%), followed by anticoagulants (87/500; 17.4%), diuretics (78/500; 15.6%) and antidepressants (78/500; 15.6%). One doctor treated the all of the patients’ diseases in 352 cases (352/500; 70.4%). Furthermore, 391 respondents claimed that they confessed the doctor to take any new medication (391/500; 78.2%). It is worth mentioning that the majority of participants bought medication without prescription (*n* = 378, 75.6%), mostly analgesics (*n* = 305, 61.0%) and vitamins (*n* = 345, 69.0%) (Table 3).

**Figure 1:**
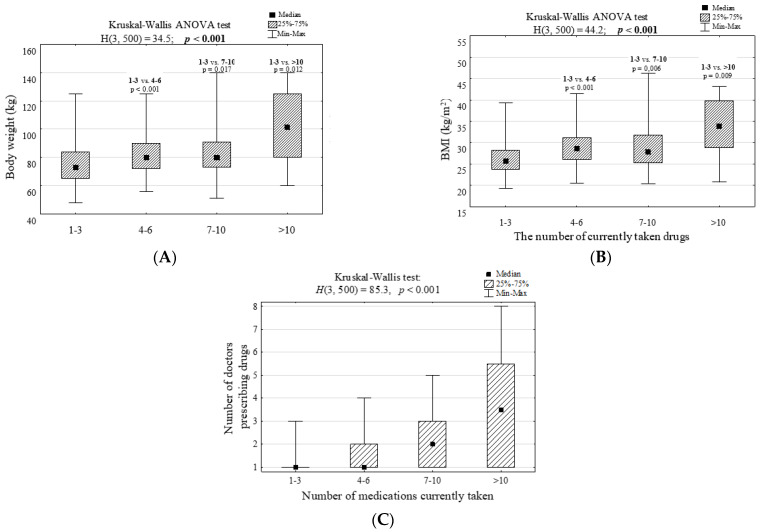
(**A**) Body weight, (**B**) body mass index and (**C**) number of doctors prescribing medicines to people differing in the number of currently taken medications and the results of significance tests.

**Figure 2:**
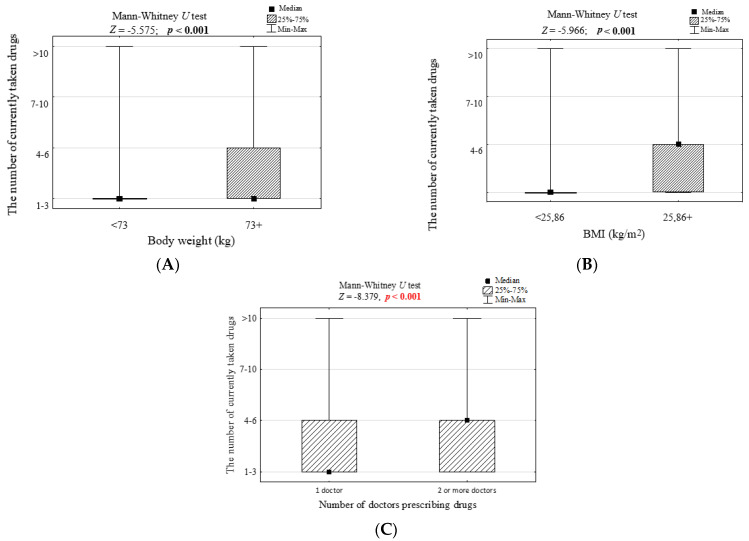
The number of drugs currently taken in groups of people differing in (**A**) body weight, (**B**) body mass index, the number of doctors who prescribed medicines (**C**) and the results of significance tests.

The study revealed no correlation (*p* > 0.05) between the number of medications taken and gender, age, multiplicity of residence, living with a household member or alone, level of education as well as net income (Table S2). However, it was observed that the greater the patient’s body mass and thus the higher the BMI, the greater the amount of medication taken (*p* < 0.001, Figure 1A,B, Table S2). Based on the Younden Index, it was also found that patients who weighed more than 73 kg and those who had BMI above 25.86 (classified as overweight) took more medications (*p* < 0.001, Figure 2A,B). It was also noted that participants who were prescribed medicines by two or more doctors used to take more medicines than those who were treated only by one doctor (*p* < 0.001, Figure 2C).

In the next section of the survey we used different scales to evaluate potential correlations between the number of currently taken drugs and the mental characteristics of elderly patients. Based on the ADL scale, it was found that patients who took more medications had a greater disability in performing basic activities of daily living (*p* = 0.017, Table S3). Furthermore, patients taking more medications had more difficulties performing complex activities (according to the IADL scale; *p* < 0.001, Table S3). Moreover, the more medications were taken, the increased likelihood to show depressive symptoms (according to GDS-15 scale; *p* < 0.001), anxiety (according to GAS-10 scale; *p* = 0.001), social isolation (according to LSNS-6 scale; *p* = 0.048), and malnutrition (according to MNA scale; *p* = 0.005, Table S3) was observed. In contrast, there was no correlation between the number of drugs taken and mental health levels (as shown on the AMTS scale) and loneliness (as shown on the Gierveld scale), Table S3.

**Figure 3:**
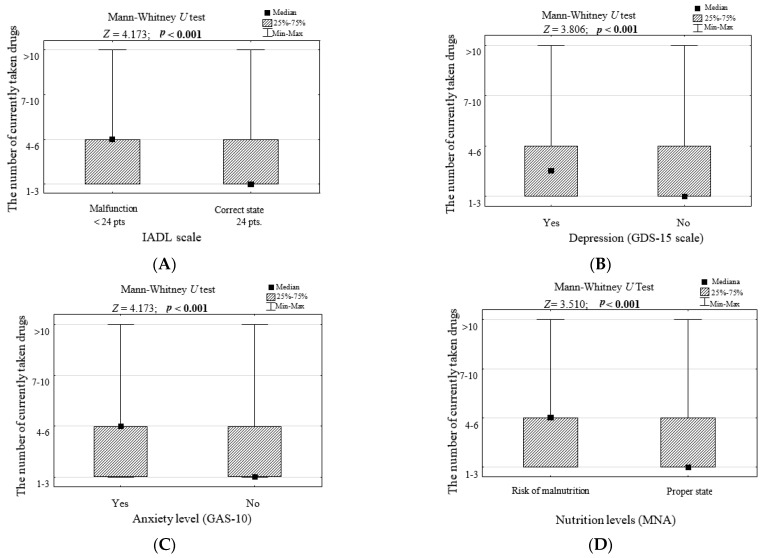
The number of medications currently taken in groups of people with different mental characteristics including (**A**) the assessment of complex activities in daily living, (**B**) depression, (**C**) the level of anxiety, (**D**) the level of malnutrition and the results of significance tests.

When classifying the respondents into groups with different mental characteristics, the criteria of the standardized tools used were adopted: disability in performing everyday activities (ADL < 5 points), depression (GDS-15 > 5 points) and the risk of malnutrition (MNA < 15 points). For complex daily life activities (IADL), elderly anxiety scores (GAS-10) and elderly social isolation scores (LSNS-6) threshold values were established based on the analysis of ROC curves (Figure S1). The areas under the curve (AUC) for the combined IADL daily activities and the assessment of anxiety in the elderly (GAS-10) are significantly greater than 0.5 (lower 95% confidence limits for AUC are greater than 0.5), which means that both these parameters have weak but statistically significant classification abilities (better than a coin toss). The parameter LSNS-6 has no classification capabilities. This statistical analysis led us to the conclusion that people with disabilities in complex daily life activities (IADL, Figure 3A), depression (GDS-15, Figure 3B), anxiety (GAS-10, Figure 3C) and the risk of malnutrition (MNA, Figure 3D) were taking significantly more medications (*p* < 0.05). The values of people with disabilities to perform basic activities of daily living (as shown in ADL < 5 points) were excluded from the analysis due to the small sample size (7/500; *p* = 0.049), which could lead to false-positive results.

Including data regarding sociodemographic, clinical, and mental characteristics of elderly patients, we found that independent predictors of taking a large number of medications per day (over 3) were (1) the presence of coronary artery disease (CAD), (2) diabetes (DM), (3) asthma (AST), (4) heart failure (HF), (5) BMI > 25.9 kg/m^2^ and (6) gastroesophageal reflux disease (GERD) (Table 4). The likelihood of taking more than three medicines per day increases approximately seven times when patients had coronary artery disease (OR = 6.77; CI 95%, 2.86–16.1). Furthermore, patients with BMI > 25,9 kg/m^2^ took more than 3 medications per day nearly three times more often than those with BMI < 25,9 kg/m^2^ (OR = 2.68, CI 95%, 1.50–4.77). Moreover, the significant correlation was also observed in participants with diabetes, asthma, heart failure and gastroesophageal reflux disease (OR = 3.23, CI 95%, 1.75–5.95; OR = 4.87, CI 95%, 2.13–11.1; OR = 3.38, CI 95%, 1.59–7.19 and OR = 1.93, CI 95%, 1.03–3.62, respectively).

The significance of the model as a whole was tested on the basis of the likelihood ratio test and Wald’s test. Chi-square = 172.6; df = 16; *p* < 0.001. A *p*-value very close to zero made us reject the null hypothesis that the model as a whole is irrelevant.

### 3.1. Antihypertensive Drugs

In the hypertension drug group, positive linear correlations with body weight (0.232), BMI (0.293), geriatric depression scale (0.104), geriatric anxiety scale (0.091), coronary artery disease (0.264), diabetes (0.228), chronic obstructive pulmonary disease (0.116), heart failure (0.250), kidney failure (0.118) have been shown. In contrast, negative linear correlations were shown by instrumental activities of daily living (−0.105) and abbreviated mental test score (−0.108) (Table S5). Based on multivariate logistic regression analysis, we found that independent predictors of anti-hypertensive drugs intake among elderly patients were AMTS < 9 pts (*p* = 0.011), CAD (*p* < 0.001), diabetes (*p* < 0.001), heath failure (HF) (*p* < 0.001) and BMI ≥ 29.0 kg/m^2^ (*p* < 0.001). Patients with BMI ≥ 29 kg/m^2^ and ATMS < 9 pts took approximately 3 times and 2 times more often anti-hypertensive drugs than other respondents (OR = 3,12, Cl 95%, 1.85–5.27 and OR = 1.70, Cl 95%, 1.04–2.78, respectively). Furthermore, patients suffering from diabetes, heart failure and coronary artery disease took approximately 3 times, 2.5 times and 4 times more often hypertension drugs than other respondents (OR = 2.88, Cl95%, 1.53–5.43, OR = 2.46, Cl 95%, 1.18–5.15 and OR = 4.05, Cl 95%, 1.79–9.21, Table 5).

### 3.2. Diuretics

In the group of diuretics drugs, a positive linear correlation was shown with body weight (0.122), BMI (0.125), coronary artery disease (0.235), diabetes (0.131), heart failure (0.157) and gastroesophageal reflux disease (0.183). In contrast, a negative linear correlation occurred in instrumental activities of daily living (−0.143) and mini nutritional assessment (−0.135) (Table S5). Based on multivariate logistic regression analysis, we found that independent predictors of diuretics intake among elderly patients were body weight (*p* < 0.001) and coronary artery disease (*p* < 0.001). Patients with body weight ≥73 kg were approximately 2.5 times more likely to take diuretics than patients < 73 kg (OR = 2.52, CI 95%, 1.21–5.26, Table 6). Furthermore, we observed a more than threefold increase in consuming diuretics among elderly patients with coronary artery disease (OR = 3.31, CI 95%, 1.64–6.68, Table 6).

### 3.3. Painkillers

The painkillers drugs group showed positive linear correlations with BMI (0.112), geriatric depression scale (0.194), geriatric anxiety scale (0.243) and asthma (0.089). In contrast, the negative linear correlation was visible with education (−0.149), net income (−0.119), Instrumental Activities of Daily Living scale (−0.123), Lubben Social Network scale (−0.094), Gierveld Loneliness Scale (−0.175) and Mini Nutritional Assessment (−0.117) (Table S4). Based on multivariate logistic regression analysis, we found that independent predictors of painkiller intake among elderly patients were BMI ≥ 25.8 kg/m^2^ (*p* = 0.028), Geriatric Anxiety Scale ≥ 9 pts (*p* < 0.001), and Gierveld Loneliness Scale GLS < 13 pts (*p* < 0.001). Patients with BMI ≥ 25.8 kg/m^2^, Geriatric Anxiety Scale ≥ 9 pts and Gierveld Loneliness Scale < 13 pts took approximately 1.5 times, 2.5 times and 2 times more often painkillers than other respondents, respectively (OR = 1.54, CI 95%, 1.04–2.29, OR = 2.59, CI 95%, 1.58–4.26 and OR = 2.08, Cl 95%, 1.38–3.13, respectively, Table 7).

### 3.4. Anticoagulants

In the group of anticoagulants, a positive linear correlation was shown with household (0.107), body weight (0.189), BMI (0.152), coronary artery disease (0.287), chronic obstructive pulmonary disease (0.175), heart failure (0.342) and gastroesophageal reflux disease (0.141). In contrast, a negative linear correlation occurred with the female sex (−0.112). Based on multivariate logistic regression analysis, we found that independent predictors of anticoagulant intake among elderly patients were body weight ≥ 81 kg (*p* < 0.001) and heart failure (*p* < 0.001). Patients with body weight ≥ 81 kg and suffering from heart failure took approximately two times and 4.5 times more often anticoagulants than other respondents (OR = 2.16, CI 95%, 1.09–4.27 and OR = 4.41, CI 95%, 2.27–8.56, respectively) (Table 8).

### 3.5. Antidepressants

Antidepressants showed a positive linear correlation with domicile (0.091), geriatric depression scale (0.264), geriatric anxiety scale (0.249) and gastroesophageal reflux disease (0.103). In contrast, the negative linear correlation was presented with the Gierveld Loneliness Scale (−0.100) and Mini Nutritional Assessment (−0.284) (Table S5). Based on multivariate logistic regression analysis, we found that independent predictors of antidepressants intake among elderly patients were living in a city of over 400,000 inhabitants (*p* = 0.008), GAS-10 > 8 pts (*p* < 0.001) and MNA < 13 pts (*p* < 0.001). Patients living in a city of over 400,000 inhabitants, with GAS-10 > 8 pts and MNA < 13 pts took antidepressants respectively approximately 2 times, 3 times and 2.5 times more often than other respondents (OR = 2.18, CI 95%, 1.20–3.94, OR = 2.91, CI 95%, 1.49–5.70 and OR = 2.64, Cl 95%, 1.54–4.53, respectively) (Table 9).

## 4. Discussion

Pharmacotherapy has become almost an inevitable element of our daily lives. Optimizing proper drug therapy for older adults remains a major challenge. According to the report by the Centers for Disease Control and Prevention 2019, approximately 83% of the US adults in their 60 s and 70 s consumed at least one prescription drug in the previous 30 days and about one-third of them used five or more medications [22]. These findings are quite similar to the Biostat report, showing that in 2019, 82.1% of the Polish population bought prescribed drugs for themselves or their relatives. This report also includes the percentage of people buying drugs available without a prescription, which was 94.6% [23]. In our study, which included 500 seniors living in Poland, all of the participants currently take at least one medication. Most of them declared taking from 1 to 3 drugs per day (301/500; 60.2%). We put efforts into identifying factors that increase the likelihood of taking more medications among the elderly population. Our results showed that patients who were treated by two or more doctors took more medicines than patients who were treated by only one doctor. This relation may appear obvious because patients with more underlying health conditions usually demand multi-specialized care and consultations with many health care providers. However, there is also a report saying that an increased number of physicians treating a patient is associated with a higher risk of unnecessary drug usage [8]. Having multiple drug prescribers is also a risk factor for drug-drug interactions and contributes to the adverse effects of polypharmacy in elderly patients [24,25]. Moreover, our results indicate that 21.8% of patients do not inform their general practitioners (GPs) about all new medicines they are taking. From this point, we would like to emphasize the need and importance of providing coordinated medical care for the patients. To obtain the necessary knowledge about patient health and treatment in use, physicians should actively reach for the necessary information by asking their patients about recent consultations with other specialists, new symptoms or changes in a treatment regimen. Comprehensive Geriatric Assessment (CGA) may also occur to be a useful tool to identify conditions with the highest priority for treatment and to optimize drug regimens in order to prevent or delay their complications. CGA was proved to be effective in decreasing the prevalence of polypharmacy and reducing the number of prescriptions and daily drug doses by deprescribing potentially inappropriate medications (PIMs). Moreover, patients who had undergone CGA had also optimized treatment by increasing the number of prescribed medications when potential prescribing omissions (PPOs) were observed. According to Unutmaz et al., the most common PIMs which were discontinued after comprehensive geriatric assessment were proton pump inhibitors, anti-dementia drugs and antipsychotics, while the most common PPOs started with vitamins D and B12 as well as antidepressants. After such interventions, the financial cost of treatment was also reduced [26,27].

According to our study, 378 surveyed people bought medications or supplements without a prescription (378/500; 75.6%). However, mixing OTC drugs with drugs prescribed by primary care physicians may handicap controlling the progress of the disease and the efficacy of the treatment. Moreover, self-medicating may potentially increase the incidence of a prescribing cascade when, unintentionally, a new drug is used to reduce the adverse effects of another drug prescribed to the patient. It is also possible that by self-medicating, patients will mask the presence of symptoms that require further investigation and defining a reason of their occurrence. Furthermore, an increased number of taken drugs enhances the likelihood of skipping medications essential for treatment or using incorrect drug dose. This is especially important in the elderly patients, who generally have more troubles with memory and concentration than their younger counterparts [28]. Abusing prescribed medications as well as a wrong dosage, increase the risk of adverse drug effects including headache, nausea and vomiting, dizziness, excessive sweating, bleeding or cognitive impairment [29]. Due to changes in body metabolism progressing with age and, as a consequence, changes in drugs pharmacokinetics and pharmacodynamics, older people often need to adjust doses of certain drugs or demand fewer daily doses, what puts them at a higher risk of adverse drug reactions. Inappropriate usage of drugs may prolong the drug’s effect and increase the risk of side effects [30].

Moreover, we observed that respondents with higher body weight and subsequent higher BMI values, representing overweight groups, were more likely to consume more medications per day (BMI > 25.9 kg/m^2^–OR = 2.68, CI 95%, 1.50–4.77), especially anti-hypertensive drugs (BMI ≥ 29.0 kg/m^2^–OR = 3.12, CI 95%, 1.85–5.27), diuretics (body weight ≥ 73 kg–OR = 2.52, CI 95%, 1.21–5.26), painkillers (BMI ≥ 25.8 kg/m^2^–OR = 1.54, CI 95%, 1.04–2.29) and anticoagulants (body weight ≥ 81 kg–OR = 2.16, CI 95%, 1.09–4.27). As obesity greatly increases the risk of different chronic disease incidence and mortality (diabetes, cardiovascular diseases, depression, certain cancers, etc.), it is reasonable that overweight people take more drugs [31]. This observation indicates the importance of adequate and broad lifestyle education and the need to maintain the proper balance between the quality and number of calories consumed with foods and beverages and patients’ energy requirements, including recommended physical activity, to prevent excessive weight gain. Such efforts will decrease the risk of chronic disease incidence and mortality and reduce the number of medications taken [32].

Surveyed patients were also asked to answer the questions regarding mental health conditions. We found that people with low mental levels are more likely to take drugs for hypertension (as shown on the AMTS scale; OR = 1.70, CI 95%, 1.04–2.78). Several studies proved that depression and hypertension share common pathways [33,34,35]. Therefore, our findings confirm these analyses; antihypertensive drug consumption testifies the existence of the disease that is additionally intensified among those with depression. Furthermore, patients with anxiety and social loneliness took more painkillers (as shown by the GAS-10 scale and Gierveld Scale; OR = 2.59, CI 95%, 1.58–4.26 and OR = 2.08, CI 95%, 1.38–3.13, respectively). Last but not least, antidepressants were taken more often among those with anxiety and the risk of malnutrition (as shown by the GAS scale and MNA scale OR = 2.91, CI 95%, 1.49–5.70, and OR = 2.64, CI 95%, 1.54–4.53). In these times of great anxiety and distress, special care should be given to evaluating mental health of all patients. Results of our study indicated that, according to the GDS-15 scale, more than one-third of the study group showed depressive symptoms (176/500; 35.2%). The prevalence of depression in our study was higher than in similar publications, which may be connected with the ongoing COVID-19 pandemic and social isolation [36,37]. Low mental levels, referred to as low energy levels, depressed mood, poor concentration, change in appetite, increase the risk of physical disorders such as stroke and other cardiovascular diseases, chronic obstructive pulmonary disease and pain [38]. Therefore, it is reasonable that patients with low mental conditions are likely to take more medications. We also found that antidepressants were more often taken among elderly patients living in a city with over 400,000 inhabitants (OR = 2.18, CI 95%, 1.20–3.94. Our result is consistent with other studies in this field, showing that the prevalence of depression and mental health disorders is higher in urban areas [39,40].

Sociodemographic and health-related factors that increase the risk of excessive consumption of drugs should be taken into account by primary care physicians who have the best possibilities to perform a coordinated care for the patient. GPs are often challenged to adjust drug prescription to the needs of each individual with regard to disease-specific clinical practice guidelines. Currently, available medications are often produced with excluded tests for older patients; they are approved in doses that may not be appropriate for older adults [41]. Many medications should be considered with caution due to the age-related changes in pharmacodynamics and pharmacokinetics (different absorption, distribution, metabolism and drug excretion). Clinical practice guidelines recommend prescribing medications for each disease. Still, in the case of older adults, it is worth using a common sense in deciding which medications should be assigned first to prevent the emergence of adverse effects and treat a particular disease [42]. In the study of Saraf et al., patients assigned after acute hospitalization to a qualified nursing facility were prescribed an average of 14 medications. One-third of them had adverse effects that could intensify the underlying geriatric syndromes [43]. Furthermore, Nightingale et al. found that among ambulatory seniors with cancer, 84% of them received five or more medications and 43% received more than 10 medications [44]. There are some tools available that may help to identify potentially inappropriate medication use, including The Beers, STOPP (Screening Tool for Older People’s Prescriptions) and START (Screening Tool to Alert to the Right Treatment). According to Whitman et al., after the usage of a three-tool assessment in patients from a geriatric oncology clinic, 73% of potentially inappropriate medications were identified and deprescribed, which led to a reduction of patients’ symptoms in 2/3 cases. Given the circumstances, deprescribing should be considered as a proper therapeutic intervention [45,46].

It is worth noting that awareness is the first step to prevent polypharmacy. A more systematic approach is required to tailor medication regimens to the needs of individuals [47]. Pharmacists and healthcare professionals should play an active role in educating patients regarding potential dangers of over-consuming medications and provide them with proper non-pharmacological interventions, which may relieve the symptoms and reduce the number of needed medications.

There are several limitations to our study. First of all, the cross-sectional nature of this study precluded any conclusion about causal relations; therefore, it is challenging to draw firm assumptions about the direction of exposure-outcome associations. Secondly, data were not obtained from medical documentation. All respondents were interviewed by computer-assisted telephone calls, which may increase the risk of potential biases (eg. social desirability bias). Furthermore, data were generated during the prevailing COVID-19 pandemic, which may lead to finding false, supposedly significant conclusions (e.g., financial status). Last but not least, to accurately represent the Polish adult population in our data, a stratified sampling per the voivodeships’ demographic structure was used. However, target quotas for sex and age strata were implemented in each geographical region. Therefore, we are aware of the inherent limitations of quota sampling.

## 5. Conclusions

Polypharmacy is a constantly increasing public health problem among the elderly population. This study proved that excessive drug intake is associated with coronary artery disease, diabetes, asthma, chronic obstructive pulmonary disease, heart failure, kidney failure and depression. Mental health conditions seem to play a significant role in the usage of antihypertensive drugs, painkillers, drugs for digestive ailments and antidepressants. Increased body weight and BMI are connected with a higher number of used medications. Our findings indicate a strong need to consider sociodemographic and health-related factors when prescribing appropriate medications for patients.

## Figures and Tables

**Table 1:** General characteristics of the studied elderly people.

Feature (Variable)	Statistics
Gender	
Women	290 (58.0%)
Men	210 (42.0%)
Age (years)	
60–64	141 (28.2%)
65–69	128 (25.6%)
70 and more	231 (46.2%)
Domicile	
Village	110 (22.0%)
City up to 20,000 inhabitants	56 (11.2%)
A city with 20,000 to 100,000 inhabitants	136 (27.2%)
A city with 100,000 to 200,000 inhabitants	62 (12.4%)
A city with 200,000 to 400,000 inhabitants	39 (7.8%)
A city with over 400,000 inhabitants	97 (19.4%)
Household size	
I live alone	108 (21.6%)
I live with my partner	202 (40.4%)
I live with my partner and our children	117 (23.4%)
I live alone with my children	35 (7.0%)
I live with a family	29 (5.8%)
A different situation	9 (1.8%)
Education	
Primary	8 (1.6%)
Vocational	105 (21.0%)
Secondary	245 (49.0%)
Higher	142 (28.4%)
Body mass (kg)	
M ± SD	78.5 ± 15.7
Me (IQR)	76 (67–88)
Min–Max	48–140
Body height (cm)	
M ± SD	169 ± 9
Me (IQR)	168 (163–175)
Min–Max	141–210
BMI (kg/m^2^)	
M ± SD	27.4 ± 4.6
Me (IQR)	27 (24–30)
Min–Max	19–46
Net income per person in the household per month	
<500 PLN	5 (1.0%)
501–1000 PLN	24 (4.8%)
1001–2000 PLN	188 (37.6%)
2001–3000 PLN	158 (31.6%)
Above 3000 PLN	110 (2.0%)
Refusal	15 (3.0%)

**Table 2:** Clinical characteristics of the studied people.

Chronic Diseases:	Statistics
Coronary artery disease	63 (12.6%)
Diabetes	74 (14.8%)
Asthma	43 (8.6%)
COPD	33 (6.6%)
Heart failure	71 (14.2%)
Kidney failure	20 (4.0%)
Physician-diagnosed gastroesophageal reflux disease (GERD)	68 (13.6%)
Vaccinations:	Statistics
He/she was vaccinated against the flu in 2019	62 (12.4%)
He/she was vaccinated against the flu in 2020	51 (10.2%)
Avoids vaccination because of possible complications	164 (32.8%)
You want to get vaccinated against the flu, but it is difficult due to the lack of a vaccine in pharmacies	104 (20.8%)
The primary care physician recommended flu and pneumococcal immunization	81 (16.2%)
He/she knows about flu vaccine reimbursement for seniors	259 (51.8%)

**Table 3:** Characteristics of pharmacological treatment of the studied persons.

Questionnaire Questions	Statistics
1. How many drugs are you currently taking?	
1–3	301 (60.2%)
4–6	151 (30.2%)
7–10	40 (8.0%)
>10	8 (1.6%)
2. Which group of medications do they belong to?	
Hypertension drugs	255 (51.0%)
Diuretics	78 (15.6%)
Painkillers	230 (46.0%)
Anticoagulants	87 (17.4%)
Antidepressants	78 (15.6%)
3. Have you been prescribed all the medications by the same doctor?	
Yes	352 (70.4%)
No	148 (29.6%)
4. How many different doctors prescribed the medications you are taking?	
1	352 (70.4%)
2	82 (16.4%)
3	52 (10.4%)
4	10 (2.0%)
5 and more	4 (0.8%)
5. Do you inform your family doctor about all new medications?	
Yes	391 (78.2%)
No	109 (21.8%)
6. Do you buy drugs and/or supplements without a prescription?	
Yes	378 (75.6%)
No	122 (24.4%)
7. Please select over-the-counter medications/supplements:	
Painkillers (paracetamol, ibuprofen, acetylsalicylic acid, metamizole, ketoprofen, diclofenac)	305 (61.0%)
Drugs for heartburn (proton pump inhibitors, for example: omeprazole, pantoprazole, etc.)	132 (26.4%)
Herbal (St. John’s wort, ginseng, Ginkgo biloba)	155 (31.0%)
Vitamins (C, B, D)	345 (69.0%)
Other (magnesium, potassium, calcium, zinc, selenium)	96 (19.2%)

**Table 4:** Results of logistic regression of univariate and multivariate sociodemographic, clinical and mental parameters with the use of more than 3 drugs a day.

Predictors of Taking More than 3 Drugs a Day	Univariate						Multivariate
	Number of Drugs				*p*	OR (95% CI)	OR (95% CI)
	4 or More *n* = 199		1–3 *n* = 301				
	*n*	%	*n*	%			
Body mass > 73 kg	150	75.4	152	50.5	<0.001	3.00 (2.02–4.45)	1.48 (0.83–2.61)
**BMI > 25.9 kg/m^2^**	**152**	**76.4**	**146**	**48.5**	**<0.001**	**3.43 (2.31–5.11)**	**2.68 (1.50–4.77)**
ADL < 5 pkt.	195	98.0	298	99.0	0.444	0.49 (0.11–2.22)	1.18 (0.16–8.62)
IADL < 24 pkt.	78	39.2	140	46.5	0.118	0.74 (0.52–1.07)	1.52 (0.92–2.50)
GDS-15 > 5 pkt.	105	52.8	144	47.8	0.315	1.22 (0.85–1.74)	1.28 (0.72–2.25)
GAS-10 > 7 pkt.	101	50.8	100	33.2	<0.001	2.07 (1.44–2.99)	1.46 (0.90–2.36)
LSND-6 < 15 pkt.	105	52.8	144	47.8	0.315	1.22 (0.85–1.74)	0.94 (0.60–1.49)
MNA < 12 pkt.	46	23.1	36	12.0	0.001	2.21 (1.37–3.57)	1.83 (0.99–3.38)
**CAD**	**54**	**27.1**	**9**	**3.0**	**<0.001**	**12.1 (5.80–25.2)**	**6.77 (2.86–16.1)**
**Diabetes**	**51**	**25.6**	**23**	**7.6**	**<0.001**	**4.17 (2.45–7.08)**	**3.23 (1.75–5.95)**
**Asthma**	**30**	**15.1**	**13**	**4.3**	**<0.001**	**3.93 (2.00–7.75)**	**4.87 (2.13–11.1)**
COPD	20	10.1	13	4.3	0.016	2.48 (1.20–5.10)	0.37 (0.14–1.02)
**Heart failure**	**56**	**28.1**	**15**	**5.0**	**<0.001**	**7.47 (4.08–13.7)**	**3.38 (1.59–7.19)**
Kidney failure	12	6.0	8	2.7	0.066	2.35 (0.94–5.86)	1.62 (0.49–5.35)
**GERD**	**39**	**19.6**	**29**	**9.6**	**0.002**	**2.29 (1.36–3.84)**	**1.93 (1.03–3.62)**

The goodness of fitting the logistic model to the data is presented using the accuracy (Table S4) and the ROC (Receiver Operating Characteristic) curve (Figure S2). Bold distinguish significant parameters at the level of *p* < 0.05.

**Table 5:** Sociodemographic, clinical and mental characteristics in groups that differ in hypertension medication intake and test results.

Feature (Variable)	He/She Is Taking Medication for High Blood Pressure				*p*-Value	OR (95% CI)	Multivariate Logistic Regression
	Yes *n* = 255		No *n* = 245				OR (95% CI)
Body weight ≥ 75 kg	169	66.3	111	45.3	<0.001	2.37 (1.65–3.41)	1.03 (0.63–1.66)
BMI ≥ 29.0 kg/m^2^	118	46.3	46	18.8	<0.001	3.73 (2.49–5.58)	**3.12 (1.85–5.27)**
IADL < 23 pts	64	25.1	40	16.3	0.020	1.72 (1.10–2.67)	1.18 (0.71–1.97)
AMTS < 9 pts	65	25.5	39	15.9	0.011	1.81 (1.16–2.81)	**1.70 (1.04–2.78)**
GDS ≥ 3 pts	173	67.8	138	56.3	0.010	1.64 (1.14–2.36)	1.20 (0.76–1.90)
GAS ≥ 6 pts	161	63.1	129	52.7	0.019	1.54 (1.08–2.20)	1.08 (0.69–1.72)
CAD	54	21.2	9	3.7	<0.001	7.04 (3.39–14.6)	**4.05 (1.79–9.21)**
Diabetes	58	22.7	16	6.5	<0.001	4.21 (2.35–7.57)	**2.88 (1.53–5.43)**
COPD	24	9.4	9	3.7	0.011	2.72 (1.24–5.99)	1.58 (0.64–3.95)
Heart failure	58	22.7	13	5.3	<0.001	5.25 (2.80–9.87)	**2.46 (1.18–5.13)**

Bold for parameters significant at *p* < 0.05.

**Table 6:** Sociodemographic, clinical and mental characteristics in groups that differ in diuretic intake and test results.

Feature (Variable)	He/She Is Taking Diuretics				*p*-Value	OR (95% CI)	Multivariate Logistic Regression
	Yes *n* = 78		No *n* = 422				OR (95% CI)
	*n*	%	*n*	%			
Body weight ≥ 73 kg	61	78.2	241	57.1	<0.001	2.69 (1.52–4.77)	**2.52 (1.21–5.26)**
BMI ≥ 25.6 kg/m^2^	57	73.1	249	59.0	0.022	1.89 (1.10–3.23)	0.92 (0.46–1.86)
IADL < 23 pts	27	34.6	77	18.2	0.002	2.37 (1.40–4.02)	1.63 (0.92–2.91)
MNA < 14 pts	55	70.5	234	55.5	0.017	1.92 (1.14–3.24)	1.73 (0.97–3.08)
CAD	24	30.8	39	9.2	<0.001	4.36 (2.44–7.82)	**3.31 (1.64–6.68)**
Diabetes	20	25.6	54	12.8	0.005	2.35 (1.31–4.21)	1.56 (0.83–2.96)
Heart failure	21	26.9	50	11.8	0.001	2.74 (1.53–4.90)	1.10 (0.54–2.26)

Bold for parameters significant at *p* < 0.05.

**Table 7:** Sociodemographic, clinical and mental characteristics in groups that differ in pain medication intake and test results.

Feature (Variable)	He/She Is Taking Painkillers				*p*-Value	OR (95% CI)	Multivariate Logistic Regression
	Yes *n* = 230		No *n* = 270				OR (95% CI)
Higher education	50	21.7	92	34.1	0.003	0.54 (0.36–0.80)	0.81 (0.51–1.27)
Net income up to 2.000 PLN	116	50.4	101	37.4	0.004	1.70 (1.19–2.43)	1.47 (0.98–2.21)
BMI ≥ 25.8 kg/m^2^	150	65.2	150	55.6	0.028	1.50 (1.04–2.15)	**1.54 (1.04–2.29)**
IADL < 24 pts	80	34.8	59	21.9	0.001	1.91 (1.28–2.83)	1.44 (0.93–2.23)
GDS ≥ 4 pts	138	60.0	120	44.4	0.001	1.88 (1.31–2.68)	0.84 (0.52–1.36)
GAS ≥ 9 pts	114	49.6	66	24.4	<0.001	3.04 (2.08–4.44)	**2.59 (1.58–4.26)**
LSNS < 12 pts	91	39.6	76	28.1	0.008	1.67 (1.15–2.43)	1.48 (0.96–2.28)
GLS < 13 pts	111	48.3	82	30.4	<0.001	2.14 (1.48–3.08)	**2.08 (1.38–3.13)**
MNA < 14 pts	149	64.8	140	51.9	0.004	1.71 (1.19–2.45)	1.07 (0.70–1.63)
Asthma	26	11.3	17	6.3	0.047	1.90 (1.00–3.59)	1.53 (0.76–3.09)

Bold for parameters significant at *p* < 0.05.

**Table 8:** Sociodemographic, clinical and mental characteristics in groups that differ in anticoagulant drug intake and test results.

Feature (Variable)	He/She is Taking Anticoagulants				*p*-Value	OR (95% CI)	Multivariate Logistic Regression
	Yes *n* = 87		No *n* = 413				OR (95% CI)
Female	40	46.0	250	60.5	0.017	0.55 (0.35–0,88)	0.88 (0.51-1.53)
Lives with a partner or family	49	56.3	182	44.1	0.044	1.64 (1.03–2.61)	1.56 (0.93–2.62)
Body weight ≥ 81 kg	50	57.5	136	32.9	<0.001	2.75 (1.72–4.41)	**2.16 (1.09–4.27)**
BMI ≥ 27.2 kg/m^2^	51	58.6	177	42.9	0.009	1.89 (1.18–3.02)	0.95 (0.49–1.84)
CAD	29	33.3	34	8.2	<0.001	5.57 (3.16–9.83)	1.97 (0.97–3.99)
COPD	14	16.1	19	4.6	<0.001	3.98 (1.91–8.29)	2.11 (0.89–5.01)
Heart failure	35	40.2	36	8.7	<0.001	7.05 (4.07–12.2)	**4.41 (2.27–8.56)**

Bold for parameters significant at *p* < 0.05.

**Table 9:** Sociodemographic, clinical and mental characteristics in groups that differ in antidepressant drug intake and test results.

Feature (Variable)	He/She Is Taking Antidepressants				*p*-Value	OR (95% CI)	Multivariate Logistic Regression
	Yes *n* = 78		No *n* = 422				OR (95% CI)
Lives in a city of over 400.000 inhabitants	24	30.8	73	17.3	0.008	2.12 (1.23–3.66)	**2.18 (1.20–3.94)**
GDS-15 ≥ 4 pts	61	78.2	197	46.7	<0.001	4.10 (2.32–7.25)	1.95 (0.96–3.94)
GAS-10 ≥ 8 pts	57	73.1	144	34.1	<0.001	5.24 (3.06–8.99)	**2.91 (1.49–5.70)**
GLS < 12 pts	19	24.4	57	13.5	0.024	2.06 (1.15–3.71)	1.11 (0.58–2.13)
MNA < 13 pts	48	61.5	129	30.6	<0.001	3.63 (2.20–6.00)	**2.64 (1.54–4.53)**

Bold for parameters significant at *p* < 0.05.

## Data Availability

The authors confirm that the data supporting the findings of this study are available within the article.

## Supplementary Materials

The following supporting information can be downloaded at: https://www.mdpi.com/article/10.3390/ijerph19148766/s1, Figure S1: ROC curves and threshold values for the assessment of complex daily life activities (IADL), the assessment of anxiety in the elderly (GAS-10) and the assessment of social isolation of the elderly (LSNS-6); Figure S2: ROC curves for the logistic model as a variable classifying the subjects into the group taking more than 3 drugs a day and the area under the curve (AUC); Table S1: Characteristics of the mental traits of the respondents; Table S2: Characteristics of pharmacological treatment of the studied persons, Table S3: Mental characteristics of the respondents in groups differing in the number of medications taken, Table S4: Match relevance table; Table S5: Linear correlation coefficients between drug groups and sociodemographic, clinical and mental characteristics.
