# Supplementary material for: Sociodemographic and Health-Related Factors Influencing Drug Intake among the Elderly Population
Source: Int J Environ Res Public Health. 2022 Jul 19;19(14):8766. doi: 10.3390/ijerph19148766 (PMC9325201; doi:10.3390/ijerph19148766)
Supplement: Supplementary file 1 [file ijerph-19-08766-s001.zip › Figure S1 (R2).pdf]

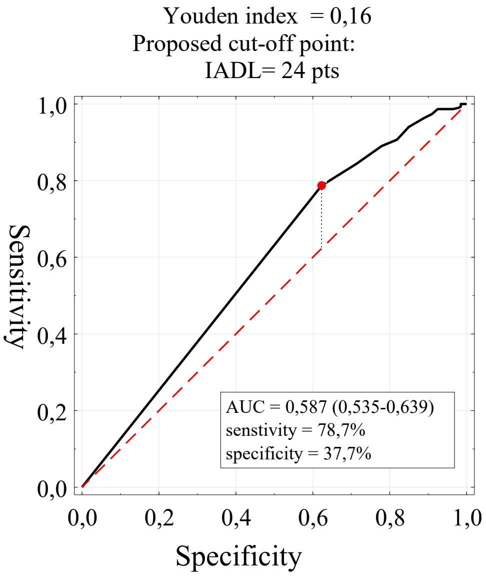

(A)

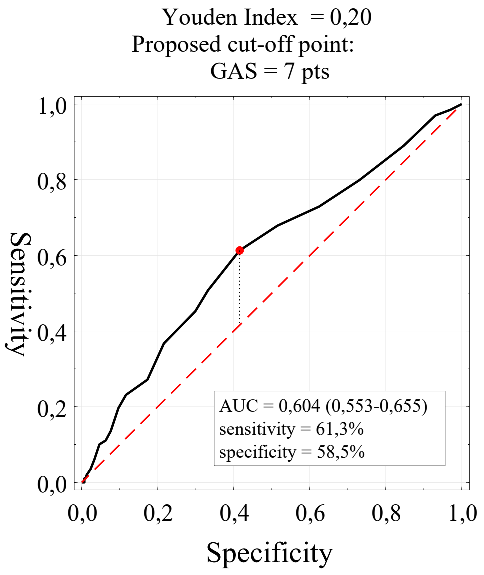

(B)

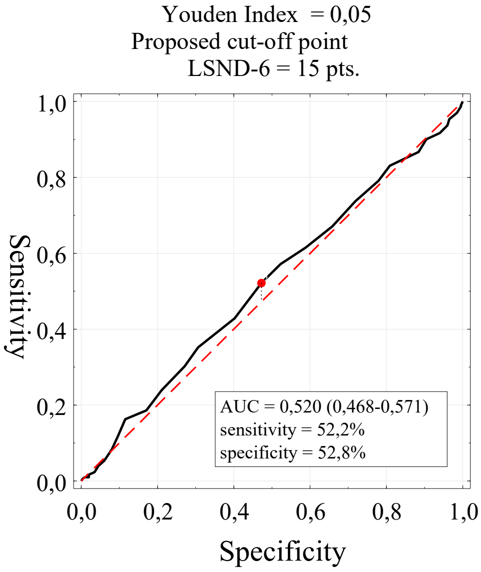

(C)

Figure S1. ROC curves and threshold values for (A)the assessment of complex daily life activities (IADL), (B)the assessment of anxiety in the elderly (GAS-10) and (c) the assessment of social isolation of the elderly (LSNS-6).
