# Supplementary material for: Sociodemographic and Health-Related Factors Influencing Drug Intake among the Elderly Population
Source: Int J Environ Res Public Health. 2022 Jul 19;19(14):8766. doi: 10.3390/ijerph19148766 (PMC9325201; doi:10.3390/ijerph19148766)
Supplement: Supplementary file 1 [file ijerph-19-08766-s001.zip › Figure S2 (R2).pdf]

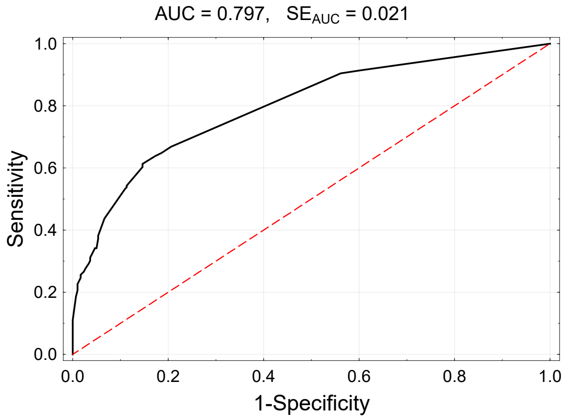

Figure S2. ROC curves for the logistic model as a variable classifying the subjects into the group taking more than 3 drugs a day and the area under the curve (AUC)
