# Supplementary material for: Sociodemographic and Health-Related Factors Influencing Drug Intake among the Elderly Population
Source: Int J Environ Res Public Health. 2022 Jul 19;19(14):8766. doi: 10.3390/ijerph19148766 (PMC9325201; doi:10.3390/ijerph19148766)
Supplement: Supplementary file 1 [file ijerph-19-08766-s001.zip › Table S1 (R2).pdf]

Table S1. Characteristics of the mental traits of the respondents

Feature (variable)	Statistics
Assessment of basic activities of daily living (ADL scale)	
M ± SD	5.9 ± 0.4
Me (IQR)	6 (6-6)
Min - Max	2 - 6
Fit people (5 - 6 points)	493 (98.6%)
Moderately disabled people (3 - 4 points)	6 (1.2%)
Disabled people (0 – 2 points)	1 (0.2%)
Assessment of Complex Daily Life Activities (IADL scale)	
M ± SD	22.9 ± 2.3
Me (IQR)	24 (23-24)
Min - Max	11 - 24
Mental performance assessment (AMTS scale)	
M ± SD	9.1 ± 1.0
Me (IQR)	9 (9-10)
Min - Max	5 - 10
Normal condition (7 - 10 points)	9.1 ± 1.0
Moderate disturbances (4 - 6 points)	9 (9-10)
Assessment of depression in the elderly (GDS-15 scale)	
M ± SD	4.8 ± 4.0
Me (IQR)	4 (2-8)
Min - Max	0 - 15
No depression (0 - 5 points)	324 (64.8%)
Depression (6 - 15 points)	176 (35.2%)
Assessment of anxiety in the elderly on the GAS-10 scale	
M ± SD	7.2 ± 4.6
Me (IQR)	6 (4-10)
Min - Max	0 - 25
Assessment of social isolation of older people (LSNS-6 scale)	
M ± SD	14.2 ± 5.9
Me (IQR)	15 (10-18)
Min - Max	0 - 30
Assessment of loneliness on the Gierveld scale	
M ± SD	13.1 ± 1.8
Me (IQR)	13 (12-14)
Min - Max	6 - 18
Nutritional Assessment (MNA scale)	
M ± SD	12.8 ± 1.5
Me (IQR)	13 (12-14)
Min - Max	6 - 14
Proper nutritional status (12 - 14 points)	418 (83.6%)
Threat of malnutrition (8 - 11 points)	78 (15.6%)
Malnutrition (0 - 7 points)	4 (0.8%)
