# Supplementary material for: Sociodemographic and Health-Related Factors Influencing Drug Intake among the Elderly Population
Source: Int J Environ Res Public Health. 2022 Jul 19;19(14):8766. doi: 10.3390/ijerph19148766 (PMC9325201; doi:10.3390/ijerph19148766)
Supplement: Supplementary file 1 [file ijerph-19-08766-s001.zip › Table S2 (R2).pdf]

Table S2. Characteristics of pharmacological treatment of the studied persons

Feature (variable)	Number of medications currently taken								P-value
	From 1 to 3 N = 301		From 4 to 6 N = 151		From 7 to 10 N = 40		More than 10 N = 8		
	n	%	n	%	n	%	n	%	
Gender									0.178
Women	182	60,5%	87	57,6%	18	45,0%	3	37,5%	
Men	119	39,5%	64	42,4%	22	55,0%	5	62,5%	
Age (years)									0.881
60 – 64	85	28,2%	41	27,2%	13	32,5%	2	25,0%	
65 - 69	77	25,6%	38	25,2%	12	30,0%	1	12,5%	
70 and more	139	46,2%	72	47,7%	15	37,5%	5	62,5%	
Domicile									0.318
Village	68	22,6%	31	20,5%	8	20,0%	3	37,5%	
City up to 20,000 inhabitants	37	12,3%	14	9,3%	3	7,5%	2	25,0%	
A city with 20,000 to 100,000	83	27,6%	44	29,1%	6	15,0%	3	37,5%	
A city with 100,000 to 200,000	33	11,0%	23	15,2%	6	15,0%	0	0,0%	
A city with 200,000 to 400,000	25	8,3%	8	5,3%	6	15,0%	0	0,0%	
A city with over 400,000 inhab.	55	18,3%	31	20,5%	11	27,5%	0	0,0%	
Household size									0.367
I live alone	69	22,9%	31	20,5%	6	15,0%	2	25,0%	
I live with my partner	110	36,5%	69	45,7%	19	47,5%	4	50,0%	
I live with my partner and our children	80	26,6%	28	18,5%	7	17,5%	2	25,0%	
I live alone with my children	22	7,3%	11	7,3%	2	5,0%	0	0,0%	
I live with a family	14	4,7%	9	6,0%	6	15,0%	0	0,0%	
A different situation	6	2,0%	3	2,0%	0	0,0%	0	0,0%	
Education									0.713
Basic	4	1,3%	3	2,0%	1	2,5%	0	0,0%	
Vocational	58	19,3%	36	23,8%	9	22,5%	2	25,0%	
Secondary	154	51,2%	64	42,4%	22	55,0%	5	62,5%	
Higher	85	28,2%	48	31,8%	8	20,0%	1	12,5%	
Net income per person									0.438
< 1000 PLN	16	5,3%	11	7,3%	2	5,0%	0	0,0%	
1001 – 2000 PLN	123	40,9%	43	28,5%	18	45,0%	4	50,0%	
2001 – 3000 PLN	88	29,2%	55	36,4%	14	35,0%	1	12,5%	
Above 3000 PLN	63	20,9%	38	25,2%	6	15,0%	3	37,5%	
Refusal	11	3,7%	4	2,6%	0	0,0%	0	0,0%	
Body weight (kg)									**<0.001**
Me (Q1-Q3)	73 (65-84)		80 (72-90)		80 (73-91)		102 (80-125)		
Min - Max	48 - 125		56 - 125		51 - 140		60 - 140		
Mass ≥ 73 kg	152	50,5%	112	74,2%	31	77,5%	7	87,5%	**<0.001**
BMI (kg/m^2)									
Me (IQR)	26 (24-28)		29 (26-31)		28 (25-32)		34 (29-40)		
Min - Max	19 - 39		21 - 42		20 - 46		21 – 43		
BMI ≥ 25,86 kg/m^2	146	48,5%	117	77,5%	28	70,0%	7	87,5%	**<0.001**
Number of doctors prescribing drugs									**<0.001**
1 doctor	252	71,6%	83	23,6%	14	4,0%	3	0,9%	
2 doctors or more	49	33,1%	68	46%	26	17,6%	5	3,4%	
