# Supplementary material for: Sociodemographic and Health-Related Factors Influencing Drug Intake among the Elderly Population
Source: Int J Environ Res Public Health. 2022 Jul 19;19(14):8766. doi: 10.3390/ijerph19148766 (PMC9325201; doi:10.3390/ijerph19148766)
Supplement: Supplementary file 1 [file ijerph-19-08766-s001.zip › Table S3 (R2).pdf]

Table S3. Mental characteristics of the respondents in groups differing in the number of medications taken

Feature (variable)	Number of medications currently taken: From 1 to 3 N = 301		From 4 to 6 N = 151		From 7 to 10 N = 40		More than 10 N = 8		P-value
Assessment of basic activities of daily living (ADL scale)									**0.017**
M ± SD	5.9 ± 0.4		5.9 ± 0.3		5.8 ± 0.5		5.5 ± 0.8		
Me (IQR)	6 (6-6)		6 (6-6)		6 (6-6)		6 (5-6)		
Min - Max	2 - 6		4 - 6		4 - 6		4 - 6		
ADL < 5 pts	3	1.0%	2	1.3%	1	2.5%	1	12.5%	**0.049**
Assessment of Complex Daily Life Activities (IADL scale)									**<0.001**
M ± SD	23.3 ± 1.8		22.6 ± 2.6		22.4 ± 2.9		19.5 ± 4.4		
Me (IQR)	24 (24-24)		24 (22-24)		24 (22-24)		20 (16-24)		
Min - Max	13 - 24		11 - 24		12 - 24		14 - 24		
IADL < 24 pts	64	21.3%	54	35.8%	16	40.0%	5	62.5%	**<0.001**
Mental performance assessment (AMTS scale)									0.273
M ± SD	9.2 ± 1.0		9.1 ± 0.9		9.0 ± 0.9		8.8 ± 1.5		
Me (IQR)	9 (9-10)		9 (9-10)		9 (8-10)		9 (8-10)		
Min - Max	5 - 10		6 - 10		7 - 10		6 - 10		
Assessment of depression in the elderly (GDS-15 scale)									**<0.001**
M ± SD	4.2 ± 3.6		5.4 ± 4.1		6.5 ± 4.2		8.4 ± 5.1		
Me (IQR)	3 (1-6)		5 (2-9)		6 (3-11)		10 (5-13)		
Min - Max	0 - 14		0 - 15		0 - 13		0 - 13		
GDS-15 > 5 pts	88	29.2%	61	40.4%	21	52.5%	6	75.0%	**0.001**
Assessment of anxiety in the elderly on the GAS-10 scale									**<0.001**
M ± SD	6.6 ± 4.4		7.8 ± 4.5		9.1 ± 5.2		10.9 ± 6.6		
Me (IQR)	6 (3-9)		7 (4-10)		9 (5-13)		12 (5-17)		
Min - Max	0 - 25		0 - 22		2 - 23		2 - 19		
GAS > 7 pts (anxiety)	100	33,2%	72	47,7%	23	57,5%	6	75,0%	**<0.001**
Assessment of social isolation of older people (LSNS-6 scale)									**0.048**
M ± SD	14.3 ± 6.0		14.2 ± 5.9		14.2 ± 5.3		9.0 ± 2.9		
Me (IQR)	15 (10-18)		14 (10-18)		15 (11-17)		10 (8-11)		
Min - Max	0 - 30		0 - 30		5 - 26		3 - 13		
LSND < 15 pts	144	47.8%	78	51.7%	19	47.5%	8	100.0%	**0.032**
Assessment of loneliness on the Gierveld scale									0.514
M ± SD	13.2 ± 1.8		13.1 ± 1.8		12.8 ± 1.6		13.8 ± 2.6		
Me (IQR)	13 (12-14)		13 (12-14)		13 (12-14)		14 (13-15)		
Min - Max	8 - 18		6 - 17		8 - 15		9 - 18		
Nutritional Assessment (MNA scale)									**0.005**
M ± SD	13.0 ± 1.2		12.5 ± 1.8		12.2 ± 1.7		11.8 ± 2.1		
Me (IQR)	13 (12-14)		13 (12-14)		12 (11-14)		12 (11-14)		
Min - Max	8 - 14		6 - 14		6 - 14		8 - 14		
MNA < 12 pts	36	12,0%	32	21,2%	11	27,5%	3	37,5%	**0.005**
