# Supplementary material for: Sociodemographic and Health-Related Factors Influencing Drug Intake among the Elderly Population
Source: Int J Environ Res Public Health. 2022 Jul 19;19(14):8766. doi: 10.3390/ijerph19148766 (PMC9325201; doi:10.3390/ijerph19148766)
Supplement: Supplementary file 1 [file ijerph-19-08766-s001.zip › Table S4 (R2).pdf]

Table S4. Match relevance table

	Predicted 1	Predicted 0	Correct
Observed 1	113	86	56.8%
Observed 0	39	262	87.0%

Odds ratio = 8.83 (5.70-13.7). Percentage of correct classifications = 75.0%
