# Supplementary material for: Sociodemographic and Health-Related Factors Influencing Drug Intake among the Elderly Population
Source: Int J Environ Res Public Health. 2022 Jul 19;19(14):8766. doi: 10.3390/ijerph19148766 (PMC9325201; doi:10.3390/ijerph19148766)
Supplement: Supplementary file 1 [file ijerph-19-08766-s001.zip › Table S5 (R2).pdf]

Table S5. Linear correlation coefficients between drug groups and socio-demographic, clinical and mental characteristics

Feature (variable)	Group of drugs							
	A	B	C	D	E	F	G	H
Female	-0.024	-0.007	-0.036	0.046	0.092	-0.112	-0.025	0.022
Age group	0.001	-0.009	0.026	-0.054	-0.030	-0.035	-0.053	-0.112
Domicile	-0.054	-0.027	-0.035	-0.060	-0.004	-0.015	0.091	-0.036
Household	0.018	0.017	0.049	0.014	0.062	0.107	0.087	0.017
Education	-0.040	-0.004	0.005	-0.149	-0.107	-0.040	-0.054	-0.011
Net income	0.015	-0.010	0.019	-0.119	-0.088	-0.052	-0.072	0.013
Body weight	0.123	0.232	0.122	0.051	-0.013	0.189	0.020	-0.078
Body Mass Index	0.162	0.293	0.125	0.112	0.043	0.152	0.029	-0.092
Activities of Daily Living	-0.086	-0.061	0.017	-0.050	-0.041	-0.023	-0.025	0.039
Instrumental Activities of Daily Living	-0.165	-0.105	-0.143	-0.123	-0.036	-0.067	-0.072	-0.025
Abbreviated mental test score	0.002	-0.108	-0.038	-0.042	-0.021	-0.060	0.014	0.081
Geriatric Depression Scale	0.157	0.104	0.063	0.194	0.156	0.060	0.264	-0.026
Geriatric Anxiety Scale	0.195	0.091	0.077	0.243	0.089	0.076	0.249	0.060
Lubben Social Network Scale	0.041	-0.012	-0.013	-0.094	-0.011	0.063	-0.077	-0.070
DeJong Gierveld Loneliness Scale	-0.077	-0.014	-0.037	-0.175	-0.076	-0.036	-0.100	-0.080
Mini Nutritional Assessment	-0.132	-0.021	-0.135	-0.117	-0.084	-0.046	-0.284	-0.039
Coronary artery disease	0.511	0.264	0.235	-0.012	0.020	0.287	-0.030	-0.035
Diabetes	0.095	0.228	0.131	-0.034	0.046	0.076	0.085	0.018
Asthma	0.108	0.072	0.084	0.089	0.077	-0.009	-0.014	0.123
Chronic obstructive pulmonary disease	0.170	0.116	0.086	0.062	-0.012	0.175	-0.048	-0.041
Heart failure	0.562	0.250	0.157	0.015	-0.021	0.342	0.015	-0.049
Kidney failure	0.017	0.118	0.081	0.037	-0.029	0.041	-0.032	0.061
Gastroesophageal reflux disease	0.093	0.039	0.183	0.067	0.268	0.141	0.103	-0.025

A - cardiac drugs, B - hypertension medications, C – diuretics, D – painkillers, E - medicines for food ailments, F – anticoagulants, G – antidepressants, H - drugs to improve memory. Correlation coefficients significant at the level of $p < 0.05$ were marked
